# A Low Frequency of IL-17-Producing CD8^+^ T-Cells Is Associated With Persistent Immune Activation in People Living With HIV Despite HAART-Induced Viral Suppression

**DOI:** 10.3389/fimmu.2018.02502

**Published:** 2018-10-29

**Authors:** Federico Perdomo-Celis, Manuel G. Feria, Natalia A. Taborda, Maria T. Rugeles

**Affiliations:** ^1^Grupo Inmunovirología, Facultad de Medicina, Universidad de Antioquia, Medellín, Colombia; ^2^Grupo de Investigaciones Biomédicas Uniremington, Programa de Medicina, Facultad de Ciencias de la Salud, Corporación Universitaria Remington, Medellín, Colombia

**Keywords:** HIV, HAART, CD8^+^ T cells, CD161, IL-17, sCD14, sulfasalazine

## Abstract

Immune activation is the hallmark of HIV infection, even in patients with highly active anti-retroviral therapy (HAART)-induced viral suppression. A major cause of immune activation during HIV infection is the intestinal microbial translocation as a consequence, among other factors, of the decrease and/or dysfunction of interleukin (IL)-17-producing T-cells, due to their role promoting the integrity of the intestinal barrier. A population of IL-17-producing CD8^+^ T-cells (Tc17 cells), characterized by the expression of CD161, has been described, but its relation with the persistent immune activation in non-viremic people living with HIV (PLWH) on HAART is unclear. By flow cytometry, we characterized the activation phenotype (evaluated by the expression of HLA-DR and CD38) of circulating CD161-expressing CD8^+^ T-cells; in addition, we explored the functionality of polyclonally-stimulated Tc17 cells in PLWH under HAART-induced viral suppression, and in healthy individuals. Finally, we determined the association of Tc17 cells with the expression of cellular and soluble activation markers. Circulating CD161-expressing CD8^+^ T-cells were decreased in PLWH compared with healthy individuals, despite their similar basal activation state. After polyclonal stimulation, IL-17 production was higher in CD8^+^ T-cells co-expressing HLA-DR and CD38 in healthy individuals. In contrast, although PLWH had a higher frequency of HLA-DR^+^ CD38^+^ CD8^+^ T-cells after stimulation, they had a lower production of IL-17. Interferon (IFN)-γ-producing CD8^+^ T-cells (Tc1 cells) were increased in PLWH. The low Tc17 cells response was associated with a high expression of CD38 and programmed death 1 protein, high levels of soluble CD14 and the treatment duration. Finally, to explore potential immunomodulatory strategies, the *in vitro* effect of the anti-inflammatory agent sulfasalazine was assessed on Tc17 cells. Interestingly, a decreased inflammatory environment, death of activated CD8^+^ T-cells, and an increased frequency of Tc17 cells were observed with sulfasalazine treatment. Thus, our findings suggest that activated CD8^+^ T-cells have a marked capacity to produce IL-17 in healthy individuals, but not in PLWH, despite HAART. This dysfunction of Tc17 cells is associated with the persistent immune activation observed in these patients, and can be partially restored by anti-inflammatory agents.

## Introduction

During human immunodeficiency virus type 1 (HIV) infection, microbial translocation, residual HIV replication or co-infections trigger and maintain the persistent inflammatory environment and the immune activation state, even in patients with viral suppression induced by the highly active anti-retroviral therapy (HAART). These alterations result in an incomplete immune reconstitution, despite continuous therapy (1, 2). As other immune cells, CD8^+^ T-cells are highly affected by the activation state, evidenced by higher levels of apoptosis, exhaustion and/or dysfunctionality (3, 4). In this sense, the expression of HLA-DR and CD38 have been widely used to assess the activation levels of CD8^+^ T-cells (5–7). Interestingly, some subpopulations have been reported based on the expression of these markers, where the CD38 expression alone and the co-expression of both molecules are associated to a higher dysfunctional state, in comparison with the unique expression of HLA-DR or the absence of both markers. Thus, HLA-DR^+^ CD38^+^ CD8^+^ T-cells from people living with HIV (PLWH) have lower cytotoxicity and non-lytic effector functions (such as the simultaneous production of cytokines) compared with HLA-DR^+^ CD38^−^ cells (8).

Based on the cytokines produced, several subpopulations of CD8^+^ T-cells, such as interferon (IFN)-γ-producing (Tc1) cells have been described. Particularly, Interleukin-17A (IL-17)-producing CD8^+^ T (Tc17) cells, characterized by the expression of CD161 (9), exhibit altered systemic frequency and function during HIV infection (10), similar to their CD4^+^ T-cells counterparts (Th17 cells) (11). The IL-17 has several beneficial effects in the intestinal tract, such as the promotion of tight junctions in epithelial cells (12), the secretion of anti-microbial peptides (13), as well as recruitment of immune cells to sites of mucosal injury (14). Therefore, during HIV infection, the lower availability of IL-17 worsens the disruption of the gut barrier and the consequent microbial translocation and immune activation.

Although HAART has contributed dramatically to decrease the acquired immunodeficiency syndrome (AIDS)-related deaths, non-AIDS conditions, with a large inflammatory component, persist in treated PLWH and are a major cause of morbidity and death in these individuals (15, 16). Thus, the search for immunomodulatory agents is one of the main research fields in the context of HAART-induced suppression of HIV replication (17). For instance, anti-inflammatory molecules such as acetylsalicylic acid, statins and hydroxychloroquine have been evaluated in PLWH on HAART to modulate the activation of monocytes (18), the expression of T-cell activation markers (19), and the reduction of inflammatory cytokines such as IL-6 and tumor necrosis factor (TNF)-α (20). In this sense, sulfasalazine (SSZ) is an anti-inflammatory agent which combines the antibiotic sulphapyridine with 5-aminosalicylic acid, and has been widely used in the treatment of rheumatoid arthritis and inflammatory bowel disease (21). Although its mechanism of action has not been completely elucidated, it has been shown to inhibit macrophage activation (22) and secretion of TNF-α (23); in addition, it induces apoptosis of activated T-cells (24). Thus, SSZ has the potential to modulate the inflammatory state in PLWH, reducing the proportion of activated and dysfunctional cells.

In this study we evaluated the frequency, phenotype and function of CD161-expressing CD8^+^ T-cells and Tc17 cells in PLWH under suppressive HAART. Overall, we hypothesized that despite HAART-induced viral suppression, PLWH have alterations in these parameters, compared with healthy controls. Certainly, despite treatment, PLWH had a decreased function of activated Tc17 cells and a low Tc17/Tc1 cells ratio compared with HIV-seronegative healthy volunteers, that correlated with high cellular and plasma immune activation levels. This dysfunction was particularly associated with the expression of CD38 and the programmed death 1 protein (PD-1). A potential beneficial effect of SSZ in the restoration of these subsets was observed. The Tc17 response is proposed as a novel cellular correlate of systemic immune activation in PLWH under HAART, as well as a potential target for the improvement of immune reconstitution in these individuals.

## Methods

### Patients and samples

The Ethical Committee of Sede de Investigación Universitaria, Universidad de Antioquia (certificates 15-08-634 and 11-08-352) approved this study. Written informed consent was obtained from all donors. All procedures followed the principles expressed in the Declaration of Helsinki. PLWH on suppressive HAART (*n* = 30) were included; all of them had a viral load <50 HIV RNA copies/mL for more than one year, reached this level in less than 26 weeks of treatment, and received only one therapeutic scheme throughout this time (56.6% receiving abacavir/lamivudine/efavirenz; 26.6% on efavirenz/emtricitabine/tenofovir, and 16.6% receiving raltegravir/tenofovir/emtricitabine). At the time of study enrollment, none of them was receiving other medications concomitantly, and none had developed therapeutic failure, AIDS-defining diseases or non-AIDS conditions, such as cardiovascular disease, neurocognitive disorders, malignancies or clinically evident co-infections. Hepatitis B or C virus co-infections were not discarded, but none of them have signs of clinical hepatitis. In all of the subjects, the mode of HIV transmission was sexual. Table 1 shows the characteristics of the study cohort. A group of HIV-seronegative healthy volunteers were included as controls (*n* = 15). To each individual, a complete medical examination and complete blood cell count was performed to exclude clinical failure (in PLWH) or disease (healthy individuals). From each individual, 10 mL of venous blood was collected in EDTA-containing tubes and the phenotyping of circulating T-cells was performed immediately. A fraction of the blood was centrifuged at 300 x g, and the plasma was used for determining viral load with the approved clinical diagnostic test RT-PCR Ampliprep-Cobas (Roche, Indianapolis, IN, USA), following the manufacturer's protocol, with a detection limit of 20 copies/mL, and for the quantification of soluble CD14 (sCD14). The cellular fraction was used for the isolation of peripheral blood mononuclear cells (PBMC). In some experiments, it was not possible to include all the individuals due to sample limitations.

**Table 1:** Characteristics of the study cohort.

**Parameter**	**Healthy (*n* = 15)**	**PLWH (*n* = 30)**	***P*-value**
Age, years; median (range)	27 (23–33)	30 (22–61)	0.1^a^
Men: Women	14: 1	30: 0	0.3^b^
CD4^+^ T-cells/μL, median (range)	634 (475–1330)	504 (428–1213)	0.009^a^
CD8^+^ T-cells/μL, median (range)	460 (174–911)	737 (268–1250)	0.001^a^
CD4:CD8 ratio, median (range)	2 (1.1–2.8)	0.7 (0.3–1.5)	<0.0001^a^
Diagnosis time, months; median (range)	N/A	26 (15–288)	N/A
Treatment time, months; median (range)	N/A	24 (14–159)	N/A

a *Mann-Whitney test*.

b *Fisher's test. N/A, Does not apply*.

### Flow cytometry

One hundred μL of whole blood was incubated for 30 min at room temperature with optimized doses and combinations of the following mouse anti-human antibodies: phycoerythrin (PE)-labeled anti-CD8 (clone RPA-T8), allophycocyanin (APC)-labeled anti-CD161 (clone DX12; both from BD, San Jose, CA, USA), Alexa Fluor 700-labeled anti-CD3 (clone UCHT1), APC-eFluor 780-labeled anti-CD4 (clone RPA-T4), fluorescein isothiocyanate (FITC)-labeled anti-HLA-DR (clone LN3), PE Cy7-labeled anti-CD38 (clone HIT2) and peridinin-chlorophyll-protein complex (PerCP)-eFluor 710-labeled anti-PD-1 (clone F38-2E2; all from Thermo Fisher, Waltham, MA, USA). Next, red blood cells were eliminated with 1X FACS Lysing Solution (BD) for 20 min at room temperature, followed by a washing step with with 1 mL of 1X PBS and fixation in 1% paraformaldehyde. The cells were acquired on a LSR Fortessa cytometer (BD), using the FACS Diva software v. 6.0, within 1 h of completing the staining; at least 50,000 CD3^+^ events were acquired. The data were analyzed with the FlowJo Software version 10.4 (Tree Star, Inc, Ashland, OR, USA). Fluorescence minus one (FMO) controls were included to define positive thresholds.

### *Ex vivo* stimulation and detection of cytokine-producing T-cells

Peripheral blood mononuclear cells were isolated using a Ficoll density gradient (Ficoll Histopaque-1077, Sigma-Aldrich, St. Louis, MO) and washed with RPMI-1640 supplemented with 10% fetal bovine serum, 100 U/mL of penicillin, 100 μg/mL of streptomycin and 2 mM L-glutamine (complete medium; all from Gibco, Carlsbad, CA). Immediately, 2 × 10^6^ cells/mL were stimulated in 96 well V-bottom plates (Costar, Corning, NY) with mouse anti-human CD28 and CD49d functional grade purified antibodies alone (both at 1 μg/mL; clones CD28.2 and 9F10, respectively, both from eBioscience; used as negative control), anti-CD28 and anti-CD49d plus a pool of HIV-1 consensus B Gag peptides (at 5 μg/mL; obtained through the NIH AIDS Reagent Program, Division of AIDS, NIAID, NIH; Cat: 8117, Lot: 140303) or with phorbol 12-myristate 13-acetate (PMA) and ionomycin (at 50 and 500 ng/mL, respectively; both from Sigma-Aldrich) and incubated for 12 h at 37 °C in 5% CO_2_, all in the presence of 5 μg/mL of Brefeldin A and monensin (both from eBioscience). After incubation, the viability was higher than 90% (assessed by Trypan blue exclusion staining). Next, the PBMC were harvested and washed with 2 mL of 1X PBS. Afterwards, the following mouse anti-human antibodies were added for cell surface staining and incubated for 30 min at 4°C, light-protected: PerCP-labeled anti-CD3 (clone SK7, BD), Alexa Fluor 700-labeled anti-CD8 (clone OKT8), APC-eFluor 780-labeled anti-HLA-DR (clone LN3) and PE-eFluor 610-labeled anti-CD38 (clone HIT2; all from Thermo Fisher). In a fraction of the individuals, APC-labeled anti-CD161 (clone DX12, BD) was also included. After cell fixation and permeabilization with Foxp3/Transcription Factor Staining Buffer Set (Thermo Fisher) and blockade with 10 μL of Fc Receptor Binding Inhibitor Polyclonal Antibody (Thermo Fisher), standardized doses of brilliant violet (BV) 510-labeled mouse anti-human IL-17A (clone N49-653, BD) and PE-Cy7-labeled mouse anti-human IFN-γ (clone 4S.B3, Thermo Fisher) were added and incubated for 30 min at 4°C, light-protected. Finally, the cells were washed twice with 1X permeabilization solution (Thermo Fisher) and acquired on a LSR Fortessa cytometer (BD). At least 25,000 CD3^+^ CD8^+^ events were acquired. Fluorescence minus one controls were also included. The background of the non-stimulated cells was subtracted from the stimulated condition. Finally, median fluorescence intensities (MeFI) of the respective markers were evaluated, ensuring comparable fluorochrome channels voltages.

### ACCENSE analysis

Automatic Classification of Cellular Expression by Nonlinear Stochastic Embedding (ACCENSE) analyses were performed to determine the phenotypic relationships from the cell populations evaluated (25), using the ACCENSE 0.0.5-beta software. Data were exported from FlowJo as FCS files and the Barnes-Hut-Stochastic Neighbor Embedding (t-SNE) was used for dimensionality reduction, with perplexity set to 30, and down sampling to 30,000 cells. t-SNE plots were colored according to the expression of chosen channel. For correlation analyses, the channels relative expression units of input were collected from a fraction of cells of all the healthy individuals and PLWH (M = 60,000 cells in each group) and computed.

### Measurement of sCD14 levels in plasma and IL-1β in culture supernatant

The Human sCD14 ELISA Kit (MyBioSource, San Diego, CA), and BD OptEIA Human IL-1β ELISA Set II (BD) were used to measure sCD14 in plasma and IL-1β in culture supernatants, following the manufacturer's protocols. The limits of detection for sCD14 and IL-1β were 0.18 ng/mL and 4.5 pg/mL, respectively.

### Sulfasalazine treatment

Peripheral blood mononuclear cells at a density of 2 × 10^6^ cells/mL were treated with 100 ng/mL of the Toll-like receptor 4 (TLR4) ligand *E. coli* lipopolysaccharide (LPS; Invivogen, San Diego, CA) in the presence or absence of 1 mM SSZ (Sigma-Aldrich, St. Louis, MO), and incubated for 24 h at 37°C in 5% CO_2_. The concentration of SSZ and the incubation time were selected after evaluation of cellular toxicity, where <1% of PBMC mortality [evaluated by Trypan blue exclusion staining and Fixable Viability Dye eFluor 506 (eBioscience)] was obtained at the chosen condition (*n* = 5, data not shown). Subsequently, the cells were stimulated with PMA and ionomycin (at 50 and 500 ng/mL, respectively), and incubated for 12 h at 37°C in 5% CO_2_, all in the presence of 5 μg/mL of Brefeldin A and monensin, previous evaluation of cell viability with the Fixable Viability Dye eFluor 506. Finally, the cells were harvested, followed by surface and intracellular staining, as described above. In a fraction of individuals, PBMC were cultured with LPS plus SSZ in the presence or absence of the caspase-1 inhibitor Ac-YVAD-cmk or the pan-caspase inhibitor Z-VAD-FMK (at 0.25 and 5 μg/mL, respectively; both from InvivoGen), and the type of cell death was evaluated with the TACS Annexin V Kits (Trevigen), following the manufacturer's instructions.

### Statistical analysis

Data are presented as medians and ranges. The Mann-Whitney and Wilcoxon tests were used for comparison of two independent or paired data, respectively, and the Kruskal-Wallis test for more than two independent groups. If the Kruskal-Wallis *P*-value was <0.05, the Dunn's multiple comparison test was performed. The degree of correlation between variables was determined with the Spearman and Pearson tests. Fisher's and Chi Square tests were used for frequency analysis. For statistical purposes, in samples with undetectable sCD14 and IL-1β levels, a value equal to the half of the assay limit of detection was assigned. In all cases, a *P*-value <0.05 was considered significant. The GraphPad Prism software v. 7.0 (GraphPad Software, La Jolla, CA) was used for the statistical analysis.

## Results

### Low frequency of circulating CD161-expressing CD8^+^ T-cells in PLWH

**Figure 1:**
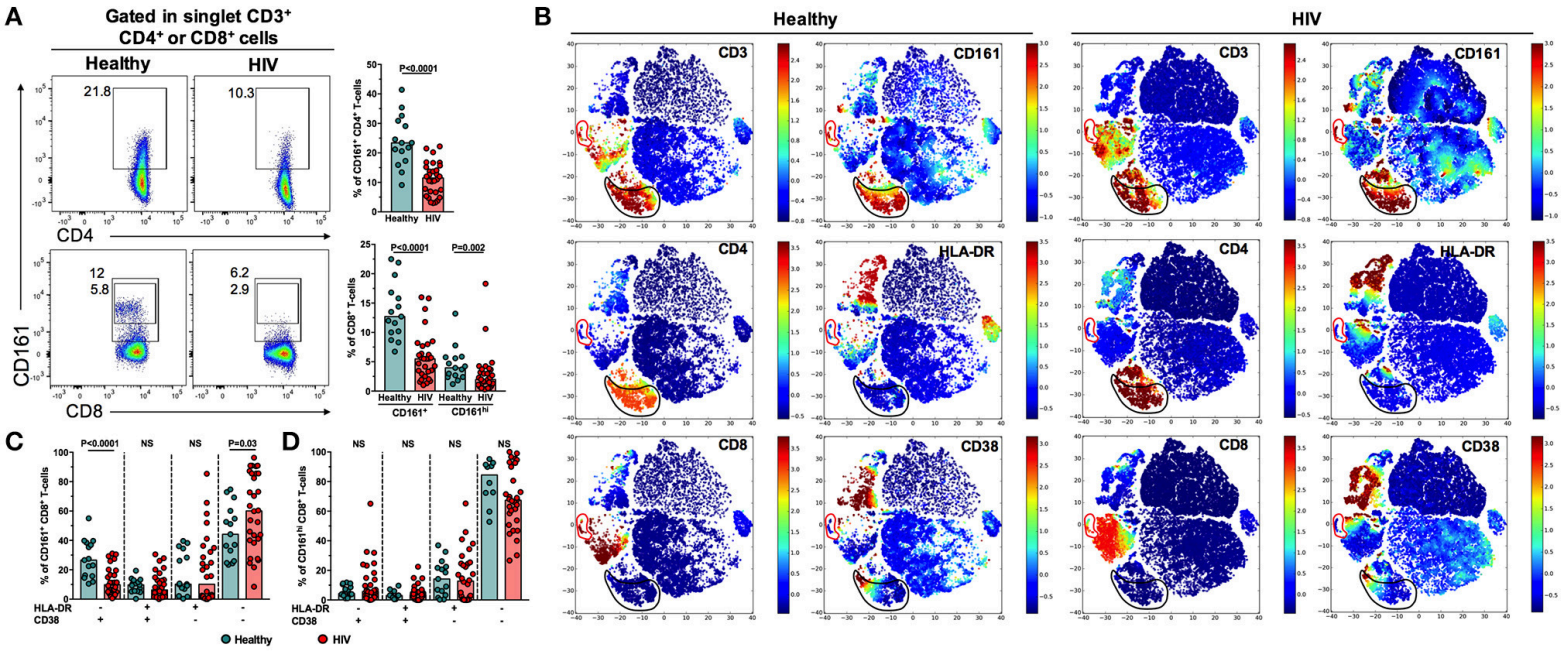
Low frequency of circulating non-activated CD161-expressing CD8^+^ T-cells in PLWH. **(A)** Gating strategy for the detection of circulating CD161-expressing CD4^+^ and CD8^+^ T-cells. Representative pseudocolor plots from a healthy individual and a subject living with HIV are shown. Peripheral blood lymphocytes were first gated and duplicates were excluded (FSC-A/FSC-H). T-cells were identified as CD3^+^. The numbers next to the gates indicate the percentage of the respective population. At the right of each plot is shown the summary of the frequency of CD161-expressing CD4^+^ and CD8^+^ T-cells in healthy individuals and PLWH. **(B)** Cell ACCENSE t-SNE plots of CD3, CD4, CD8, CD161, HLA-DR, and CD38 expression in cells from a representative healthy individual and a subject living with HIV (*M* = 30,000). The black and red circles indicate, respectively, CD161-positive CD4^+^ and CD8^+^ T-cells. **(C,D)** Frequency of HLA-DR- and/or CD38-expressing CD161^+^ **(C)** and CD161^hi^ **(D)** CD8^+^ T-cells in healthy individuals and PLWH. In **(A,C,D)** the *P*-value of the Mann-Whitney test is shown. NS, Not statistically significant.

CD161 was used as a surrogate marker for Th17 and Tc17 cells, as previously reported (10) and following the gating strategy showed in Figure 1A. Similar to a previous report (26), CD161^+^ and CD161^hi^ CD8^+^ T-cells, but not CD4^+^ T-cells, were identified, based on a 2-fold difference in the MeFI of CD161 between these subsets. The relative frequency of these cell populations (Figure 1A), as well as MeFI of CD161 (P ≤ 0.001, data not shown) were decreased in PLWH in comparison with healthy controls. To explore the activation profile of these subsets, the expression of HLA-DR and CD38, classically used to assess T-cell activation, was evaluated by ACCENSE t-SNE analysis. As shown in Figure 1B, t-SNE plots of all collected parameters in cells from a healthy individual and a subject living with HIV showed association of CD161 with CD3 and CD4 expression, and in a lesser extent with CD8 expression. Interestingly, CD161-positive cells were not related to those expressing HLA-DR and CD38, in either CD4^+^ or CD8^+^ T-cells. In fact, CD161^+^ (Figure 1C) and CD161^hi^ (Figure 1D) CD8^+^ T-cells predominantly lacked the expression of both activation markers. Non-significant differences were observed in HLA-DR and/or CD38-expressing CD161-expressing CD8^+^ T-cell populations between healthy controls and PLWH, except for a lower frequency of HLA-DR^+^ CD38^−^ CD161^+^ CD8^+^ T-cells in PLWH (Figure 1C). Together, these results indicate that, despite HAART, a lower frequency of non-activated CD161-expressing CD8^+^ T-cells is found in PLWH.

### Compared with healthy controls, PLWH under HAART-induced viral suppression have conserved total Tc17 cells

**Figure 2:**
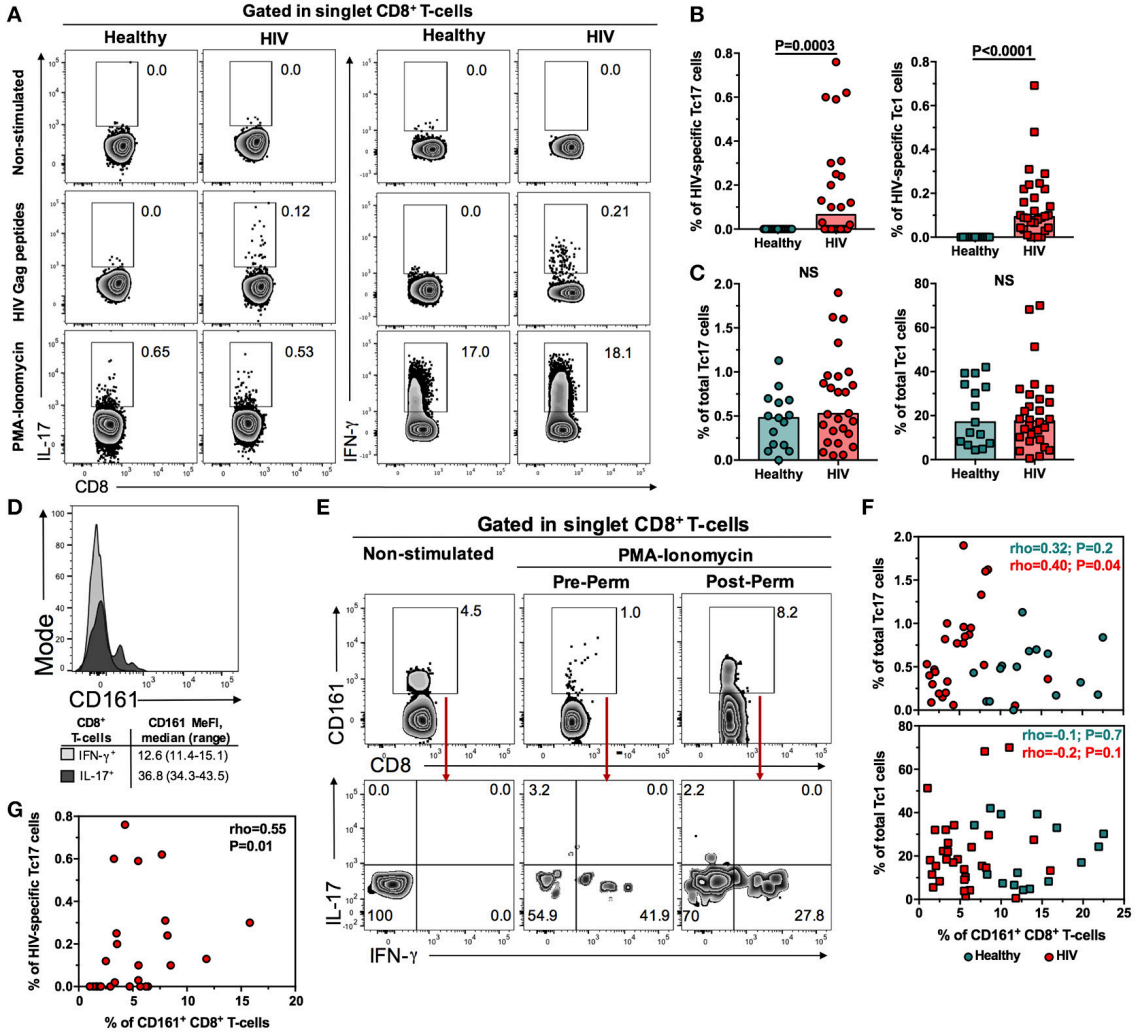
Conserved frequency of total Tc17 cells in PLWH on suppressive HAART. **(A)** Gating strategy for the detection of total and HIV-specific Tc17 and Tc1 cells. Representative zebra plots from non-stimulated, HIV Gag peptides- and PMA-ionomycin-treated cells from a healthy individual and a subject living with HIV are shown. After duplicates exclusion (FSC-A/FSC-H), CD8^+^ T-cells were identified as CD3^+^ CD8^+^ cells. The numbers next to the gates indicate the percentage of the population. **(B)** Frequency of HIV-specific Tc17 and Tc1 cells in healthy individuals and PLWH. **(C)** Frequency of total Tc17 and Tc1 cells in healthy individuals and PLWH. **(D)** Representative expression of CD161 in IL-17^+^ and IFN-γ^+^ CD8^+^ T-cells from a healthy individual, after PMA-ionomycin stimulation. The summary of the median fluorescence intensity (MeFI) of the populations is shown below. **(E)** Gating strategy for the detection, after cell culture, of IL-17- and IFN-γ-producing CD161-expressing CD8^+^ T-cells from a subject living with HIV. In the left zebra plots are shown non-stimulated cells. In the middle and right zebra plots are shown PMA-ionomycin-stimulated cells where the anti-CD161 antibody was added before (middle panels) or after (right panels) cell permeabilization. The numbers indicate the percentage of the populations. **(F)** Correlation between the frequency of total Tc17 (upper panel) and Tc1 (lower panel) cells and the frequency of circulating CD161^+^ CD8^+^ T-cells in healthy individuals and PLWH. **(G)** Correlation between the frequency of HIV-specific Tc17 cells and the frequency of circulating CD161^+^ CD8^+^ T-cells in PLWH. In **(G,H)** the rho and P value of the Spearman test are shown. NS, Not statistically significant.

To confirm the findings obtained with the analysis of CD161, we directly assessed the frequency of HIV-specific and total Tc17 cells after Gag peptides and PMA-ionomycin stimulation, respectively, following the gating strategy of Figure 2A. The frequency of Tc1 cells was also evaluated (Figure 2A). As shown in Figure 2B, a low, but detectable, frequency of HIV-specific Tc17 and Tc1 cells was found in PLWH, with no differences between both subsets (*P* = 0.4, data not shown). As expected, we did not detect HIV-specific Tc17 or Tc1 cells in healthy individuals (Figures 2A, B). The frequency of total Tc17 and Tc1 cells was similar between PLWH and healthy controls (Figure 2C). In addition, we did not observe differences in the MeFI of IL-17 and IFN-γ in total and HIV-specific Tc17 and Tc1 cells, respectively (P≥0.1, data not shown). Similarly, a comparable total Tc17/Tc1 cells ratio (as a measure of the predominant CD8^+^ T-cell subset) was found between healthy individuals and PLWH (*P* = 0.1, data not shown).

To determine the significance of the lower frequency of CD161-expressing CD8^+^ T-cells but conserved frequency of total Tc17 cells, the expression of CD161 was evaluated in IL-17^+^ and IFN-γ^+^ CD8^+^ T-cells. As shown in Figure 2D, and similar to a previous report (9), IL-17^+^ CD8^+^ T-cells from healthy individuals had a higher expression of CD161 than IFN-γ^+^ CD8^+^ T-cells. Nonetheless, we extensively observed a decrease in the frequency of CD161-expressing CD8^+^ T-cells after polyclonal stimulation (Figure 2E). In order to evaluate if CD161 was internalized after stimulation, we evaluated its expression when the anti-CD161 antibody was added pre and post cell permeabilization. Post-permeabilized stained cells had a higher frequency of CD161-expressing CD8^+^ T-cells compared with pre-permeabilized stained cells, and they produced both IL-17 and IFN-γ (Figure 2E). In extent, when we correlated the frequency of total Tc17 with that of CD161^+^ CD8^+^ T-cells, a low and lack of correlation was found in PLWH and healthy individuals, respectively (Figure 2F, upper panel), similar to the equivalent comparison with total Tc1 cells (Figure 2F, lower panel). On the contrary, a positive correlation between the frequency of HIV-specific Tc17 cells and that of CD161^+^ CD8^+^ T-cells was found (Figure 2G). In summary, although the frequency of CD161-expressing CD8^+^ T-cells is decreased in PLWH on HAART, the total Tc17 response is maintained. In addition, the expression of CD161 is not a good surrogate marker of total Tc17 cells after PMA-ionomycin stimulation because it is internalized with these potent stimuli. In the case of a less potent stimulation, such as HIV peptides, a low correlation between HIV-specific Tc17 cells response and CD161 expression was observed.

### Among total CD8^+^ T-cells, those with an activated phenotype are the main source of IL-17, and are dysfunctional in PLWH

**Figure 3:**
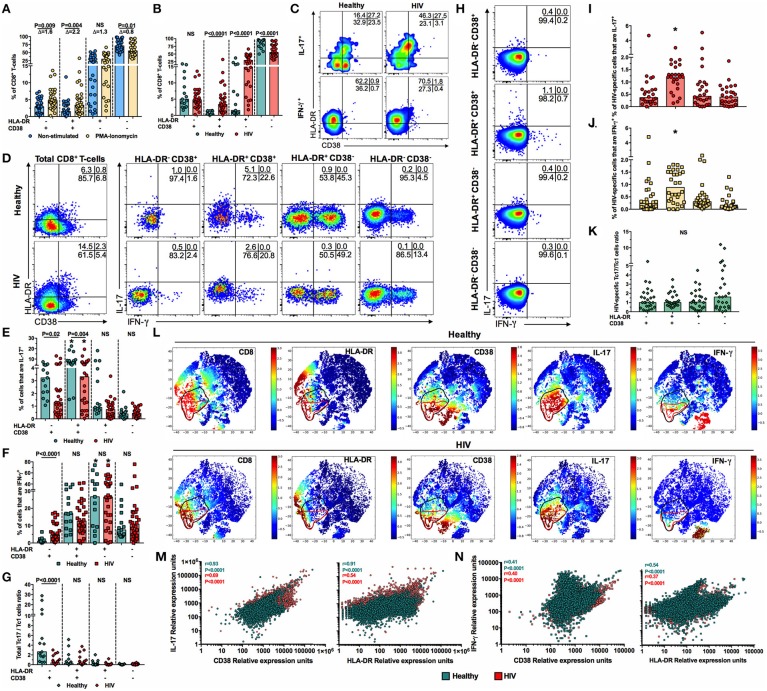
Low frequency of activated Tc17 cells in PLWH. **(A)** Frequency of HLA-DR- and/or CD38-expressing non-stimulated and PMA-ionomycin-stimulated CD8^+^ T-cells from PLWH. The Δ of the PMA-ionomycin/non-stimulated cells frequency is shown at the top. The *P*-value of the Wilcoxon test is shown. **(B)** Frequency of HLA-DR- and/or CD38-expressing PMA-ionomycin-stimulated CD8^+^ T-cells from healthy individuals and PLWH. **(C)** Expression of HLA-DR and CD38 in IL-17^+^ and IFN-γ^+^ CD8^+^ T-cells after PMA-ionomycin stimulation in a representative healthy individual and patient living with HIV. **(D)** Representative pseudocolor plots of the expression of IL-17 and IFN-γ in HLA-DR/CD38-expressing CD8^+^ T-cells after PMA-ionomycin stimulation in a representative healthy individual and patient living with HIV. **(E,F)** Frequency of IL-17^+^ **(E)** and IFN-γ^+^ **(F)** cells among HLA-DR- and/or CD38-expressing PMA-ionomycin-stimulated CD8^+^ T-cells from healthy individuals and PLWH. *Indicate which population, according to the expression of HLA-DR and CD38, had the highest frequency of IL-17^+^ or IFN-γ^+^ cells, respectively, when healthy individuals or PLWH were analyzed independently (*P* ≤ 0.02, Dunn's *post-hoc* test). **(G)** HLA-DR and/or CD38-expressing total Tc17/Tc1 cells ratio in healthy individuals and PLWH. **(H)** Representative pseudocolor plots of the expression of IL-17 and IFN-γ in HLA-DR/CD38-expressing CD8^+^ T-cells after Gag peptides stimulation in a representative patient living with HIV. **(I,J)** Frequency of IL-17^+^ **(I)** and IFN-γ^+^ **(J)** cells among HLA-DR- and/or CD38-expressing Gag-stimulated CD8^+^ T-cells from PLWH. *P ≤ 0.04 vs. all the groups; Dunn's *post-hoc* test. **(K)** HLA-DR and/or CD38-expressing HIV-specific Tc17/Tc1 cells ratio in PLWH. **(L)** Cell ACCENSE t-SNE plots of CD8, HLA-DR, CD38, IL-17, and IFN-γ expression in polyclonally-stimulated cells from a representative healthy individual and a subject living with HIV (*M* = 30,000). The black and red circles indicate, respectively, IL-17- and IFN-γ-positive CD8^+^ T-cells. **(M,N)** Correlation between the IL-17 **(M)** and IFN-γ **(N)** relative expression units and that of CD38 and HLA-DR. A total of 30,000 cells were collected from all the healthy individuals and PLWH, and computed. The r and *P*-value of the Pearson test are shown. In **(B,E–G)** the *P*-value of the Mann-Whitney test is shown. NS, Not statistically significant.

Although CD8^+^ T-cells from PLWH on HAART exhibited a low activation profile, it is possible that their activation threshold is lower than that of CD8^+^ T-cells from healthy individuals. Thus, we evaluated the expression of HLA-DR and CD38 in CD8^+^ T-cells in the presence or absence of PMA-ionomycin. Similar to previous reports (27, 28), the frequency of HLA-DR^+^ and/or CD38^+^ CD8^+^ T-cells increased after polyclonal stimulation, with the decrease of double negative cells, both in PLWH and healthy individuals (Figure 3A and data not shown). Of note, HLA-DR^+^ and CD38^+^ cells increased the most after stimulation, evidenced by the delta of the PMA-ionomycin/non-stimulated cells frequency (Figure 3A); however, their proportion, as well as that of HLA-DR^+^ CD38^−^ cells, were higher in PLWH (Figure 3B). Thus, CD8^+^ T-cells from PLWH are prone to activation after *ex vivo* polyclonal stimulation.

To explore if the higher activation levels post-stimulation in CD8^+^ T-cells from PLWH affected their ability to produce IL-17, we evaluated the magnitude of total HLA-DR^+^ and/or CD38^+^ Tc17 cells. In healthy individuals and PLWH, a high percentage of IL-17^+^ cells expressed the HLA-DR^+^ CD38^+^ phenotype (Figure 3C). In contrast, most of IFN-γ cells exhibited the HLA-DR^+^ CD38^−^ phenotype (Figure 3C). Similarly, HLA-DR^+^ CD38^+^ CD8^+^ T-cells had the highest production of IL-17 among total CD8^+^ T-cells when compared with HLA-DR^−^ CD38^+^, HLA-DR^+^ CD38^−^ and HLA-DR^−^ CD38^−^ cells (Figures 3D,E). Nonetheless, despite the higher frequency of HLA-DR^+^ CD38^+^ CD8^+^ T-cells in PLWH, their production of IL-17 cells was lower, as well as that of HLA-DR^−^ CD38^+^ cells (Figures 3D,E). On the other hand, HLA-DR^+^ CD38^−^ cells were the major producers of IFN-γ among HLA-DR/CD38-expressing CD8^+^ T-cells, and the frequency of this subset was similar between PLWH and healthy controls (Figures 3D,F). Interestingly, the total Tc17/Tc1 cells ratio, according to the expression of HLA-DR and CD38, was decreased in PLWH in comparison with healthy controls in HLA-DR^−^ CD38^+^ cells (Figure 3G). Similar to total Tc17 cells, HIV-specific HLA-DR^+^ CD38^+^ CD8^+^ T-cells exhibited the highest production of IL-17 (Figures 3H,I), similar to Tc1 cells (Figures 3H,J), with comparable frequencies between both subsets, as evidenced by a HIV-specific Tc17/Tc1 cells ratio near to 1 in all the subsets, without significant differences between them (Figure 3K).

To further confirm the findings obtained with polyclonally-stimulated cells, we performed an ACCENSE analysis to explore the relationship between the activation markers with the expression of IL-17 and IFN-γ in CD8^+^ T-cells. The t-SNE analysis showed that, in healthy individuals, the expression of IL-17 was particularly related with HLA-DR expression, and in a lesser extent with CD38 expression, while this association was not evidenced with IFN-γ-positive cells (Figure 3L, upper panels). These associations were confirmed when the relative expression of IL-17, HLA-DR and CD38 were correlated, as a high positive correlation was found between these parameters (Figure 3M). In contrast, a low correlation was found between the relative expression of IFN-γ, HLA-DR, and CD38 (Figure 3N). When these analyses were performed in PLWH, similar associations between the expression of IL-17, HLA-DR, and CD38 were found, but at a lower degree than in healthy individuals (Figures 3L,M), as well as for IFN-γ expression (Figures 3L,N). Interestingly, the ACCENSE analysis also demonstrated the association between the expression of IL-17 and IFN-γ, particularly in PLWH (Figure 3L). Nevertheless, by flow cytometry analysis we did not detect total, CD161^+^ or activated CD8^+^ T-cells co-expressing IL-17 and IFN-γ in any of the studied groups (Figures 2E, 3D,H). Taken together, our results indicate that, among total CD8^+^ T-cells, HLA-DR^+^ CD38^+^ cells are the main source of IL-17 in healthy individuals, but not in PLWH, despite their higher frequency after polyclonal activation.

### The low response of HLA-DR^+^ CD38^+^ Tc17 cells in PLWH is associated with the expression of CD38 and PD-1

**Figure 4:**
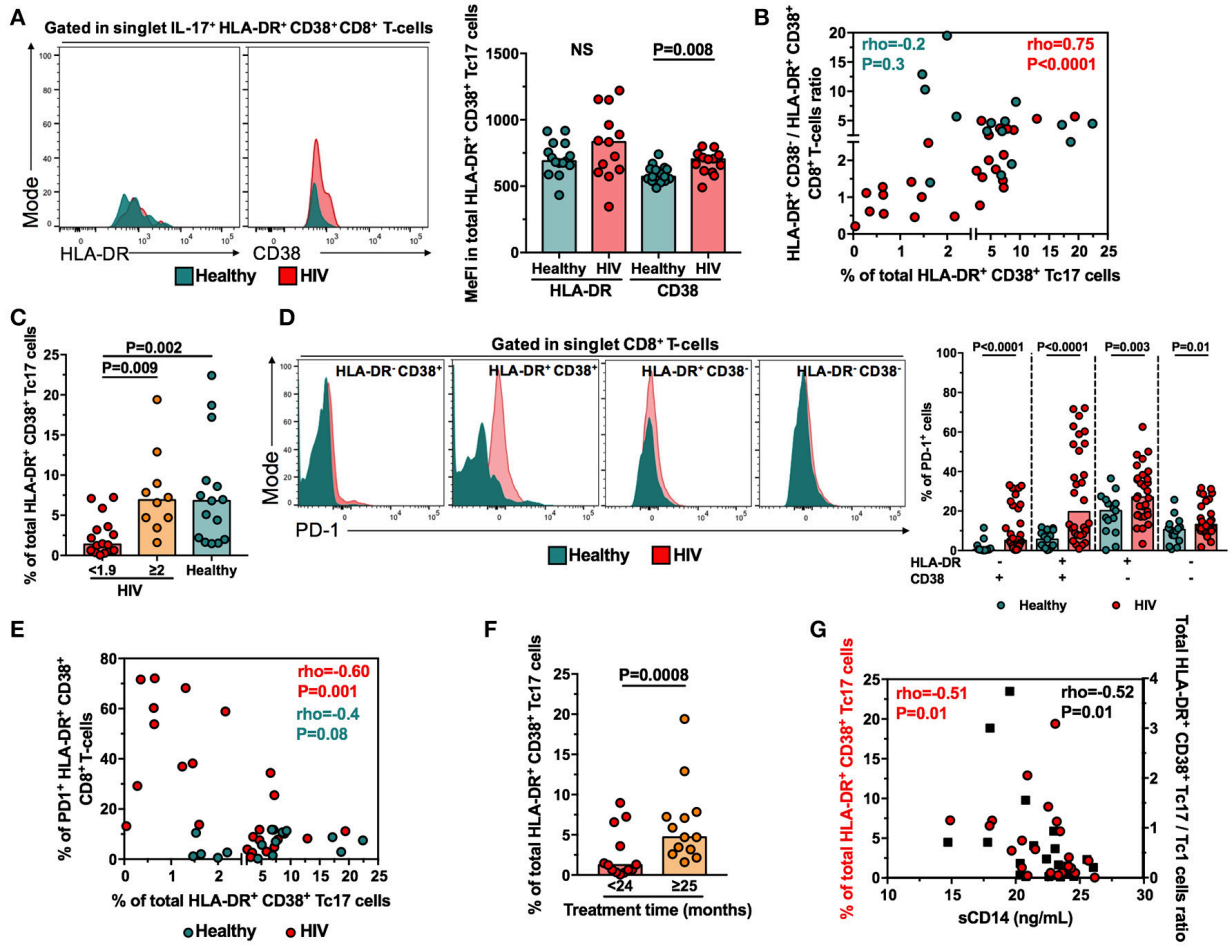
The frequency of HLA-DR^+^ CD38^+^ Tc17 cells is associated with the CD38 and PD-1 expression, treatment duration and the levels of plasma sCD14 in PLWH. **(A)** Expression of HLA-DR and CD38 in HLA-DR^+^ CD38^+^ Tc17 cells from healthy individuals and PLWH. In the right panel is shown the summary of the individuals evaluated. **(B)** Correlation between the frequency of total HLA-DR^+^ CD38^+^ Tc17 cells and the HLA-DR^+^ CD38^+^/HLA-DR^+^ CD38^−^ CD8^+^ T-cells ratio. **(C)** Frequency of HLA-DR^+^ CD38^+^ Tc17 cells in PLWH with HLA-DR^+^ CD38^+^/HLA-DR^+^ CD38^−^ CD8^+^ T-cells ratio <1.9 or ≥2, as well as healthy individuals. The *P*-value of the Kruskal-Wallis and Dunn's *post-hoc* test are shown. **(D)** Expression of PD-1 in HLA-DR and/or CD38-expressing CD8^+^ T-cells from healthy individuals and PLWH. In the right panel is shown the frequency of PD-1^+^ HLA-DR and/or CD38-expressing CD8^+^ T-cells in healthy individuals and PLWH. **(E)** Correlation between the frequency of total HLA-DR^+^ CD38^+^ Tc17 cells and the frequency of PD-1^+^ HLA-DR^+^ CD38^+^ CD8^+^ T-cells in healthy individuals and PLWH. **(F)** Frequency of HLA-DR^+^ CD38^+^ Tc17 cells in PLWH who received HAART for <24 or ≥25 months. **(G)** Correlation between the frequency of total HLA-DR^+^ CD38^+^ Tc17 cells (red circles), the total HLA-DR^+^ CD38^+^ Tc17/Tc1 cells ratio (black squares) and the levels of plasma sCD14. In **(A,D,F)** the *P*-value of the Mann-Whitney test is shown. In **(B,E,G)** the rho and *P*-value of the Spearman test are shown. NS, Not statistically significant.

Since the proportion of total HLA-DR^+^ CD38^+^ Tc17 cells is decreased in PLWH despite the higher frequency of total HLA-DR^+^ CD38^+^ CD8^+^ T-cells after polyclonal stimulation, we asked which factors differentiate HLA-DR^+^ CD38^+^ Tc17 cells from healthy individuals and PLWH. Since the unique expression of CD38, but not HLA-DR, has been associated to CD8^+^ T-cells dysfunctionality (8), we hypothesized that the density of expression of CD38 influenced the response of HLA-DR^+^ CD38^+^ Tc17 cells in PLWH. Certainly, HLA-DR^+^ CD38^+^ Tc17 cells from PLWH had a higher MeFI of CD38, but not of HLA-DR, than healthy individuals (Figure 4A). On the other hand, HLA-DR^−^ CD38^+^ Tc17 cells from healthy individuals and PLWH did not show differences in the expression of both activation markers (*P* ≥ 0.6, data not shown), suggesting that the higher expression of CD38 in PLWH is restricted to HLA-DR^+^ CD38^+^ Tc17 cells.

In addition, we analyzed the HLA-DR^+^ CD38^−^/HLA-DR^+^ CD38^+^ CD8^+^ T-cells ratio, as a measure of the predominant cell subset in PLWH. Of note, this ratio was lower in PLWH in comparison with healthy individuals [median [range] of 1.3 [0.2–5.6] and 3.7 [1.3–10.2] in PLWH and healthy individuals, respectively; *P* = 0.003]. Interestingly, a positive correlation was obtained between the frequency of total HLA-DR^+^ CD38^+^ Tc17 cells and the HLA-DR^+^ CD38^−^/HLA-DR^+^ CD38^+^ CD8^+^ T-cells ratio in PLWH, but not in healthy individuals (Figure 4B). In addition, when the PLWH were classified according to their HLA-DR^+^ CD38^−^/HLA-DR^+^ CD38^+^ CD8^+^ T-cells ratio (<1.9 and ≥2) and compared with healthy individuals, those PLWH with a ratio <1.9, but not those with a ratio ≥2, had significantly lower frequencies of HLA-DR^+^ CD38^+^ Tc17 cells (Figure 4C). Of note, these findings were not observed for total HLA-DR^−^ CD38^+^, HLA-DR^+^ CD38^−^ or HLA-DR^−^ CD38^−^ Tc17 cells (P≥0.1, data not shown). Thus, the magnitude of total HLA-DR^+^ CD38^+^ Tc17 cells is influenced by the expression of CD38, and patients with predominant HLA-DR^+^ CD38^−^ CD8^+^ T-cells response conserve their frequencies of functional Tc17 cells.

Activated CD8^+^ T-cells usually also exhibit an exhaustion state, which is widely evaluated by the expression of the inhibitory receptor PD-1; this activation/exhaustion state of CD8^+^ T-cells has been associated with disease progression in HIV infection (4). Thus, we explored if the expression of PD-1 is associated with the dysfunction of Tc17 cells. Certainly, HLA-DR^+^ CD38^+^ and HLA-DR^−^ CD38^+^ CD8^+^ T-cells in peripheral blood from PLWH had higher MeFI of PD-1 than those from healthy individuals, as well as higher percentages of PD-1^+^ cells (Figure 4D). Strikingly, the frequency of PD1^+^ HLA-DR^+^ CD38^+^ CD8^+^ T-cells negatively correlated with the frequency of total HLA-DR^+^ CD38^+^ Tc17 cells in PLWH but not in healthy individuals (Figure 4E). Together, these results indicate that the activation/exhaustion state of CD8^+^ T-cells and the inhibitory effect of PD-1, persistently increased during HIV infection despite HAART-induced viral suppression, is associated with a low Tc17 cells response in PLWH.

### The low response of HLA-DR^+^ CD38^+^ Tc17 cells is associated with the treatment duration and systemic immune activation

We analyzed the association between the frequency of activated Tc17 cells and several parameters, such as CD4^+^ and CD8^+^ T-cells count, CD4:CD8 ratio, HAART scheme received, treatment duration, and plasma sCD14, as a systemic activation marker (29). Interestingly, a higher frequency of total HLA-DR^+^ CD38^+^ Tc17 cells, but not HLA-DR^−^ CD38^+^, HLA-DR^+^ CD38^−^ and HLA-DR^−^ CD38^−^ cells, was found in patients receiving HAART for more than 25 months in comparison with those with <24 months of therapy (Figure 4F and data not shown), suggesting that the increase of this subset is associated to treatment duration. On the contrary, we did not observe significant differences when the frequency of Tc17 cells was analyzed according to CD4^+^ and CD8^+^ T-cells count, CD4:CD8 ratio and the HAART scheme received (*P* > 0.4, data not shown). Moreover, PLWH had higher levels of sCD14 than healthy controls [median [range] of 21.5 ng/mL [14.8–26.1] and 0.55 ng/mL [0.18–2.4] in PLWH and healthy individuals, respectively; *P* < 0.0001, data not shown]. Interestingly, a significant negative correlation was found between the frequency of total HLA-DR^+^ CD38^+^ Tc17 cells, the total HLA-DR^+^ CD38^+^ Tc17/Tc1 cells ratio and plasma sCD14 levels (Figure 4G). Thus, the low response of activated Tc17 cells, and a shift from Tc17 to Tc1 profile is associated with an increased immune activation state in PLWH, despite the suppression of HIV replication by HAART.

### SSZ increases the frequency of HLA-DR^+^ CD38^+^ Tc17 cells from PLWH on HAART

To explore a potential immunomodulatory strategy for the improvement of Tc17 cells response in PLWH under HAART-induced viral suppression, we evaluated the anti-inflammatory agent SSZ. Based on a previous report (24), we analyzed the effect of SSZ on the decrease of the inflammatory environment and the induction of death of activated T-cells, as well as its effect on the production of IL-17 by CD8^+^ T-cells. To reproduce *in vitro* the microbial translocation and the inflammatory environment, PBMC were treated for 24 h with LPS to induce IL-1β secretion by innate cells, in the presence or absence of SSZ, and subsequently stimulated for 12 h with PMA and ionomycin to induce the production of IL-17 and IFN-γ by CD8^+^ T-cells.

**Figure 5:**
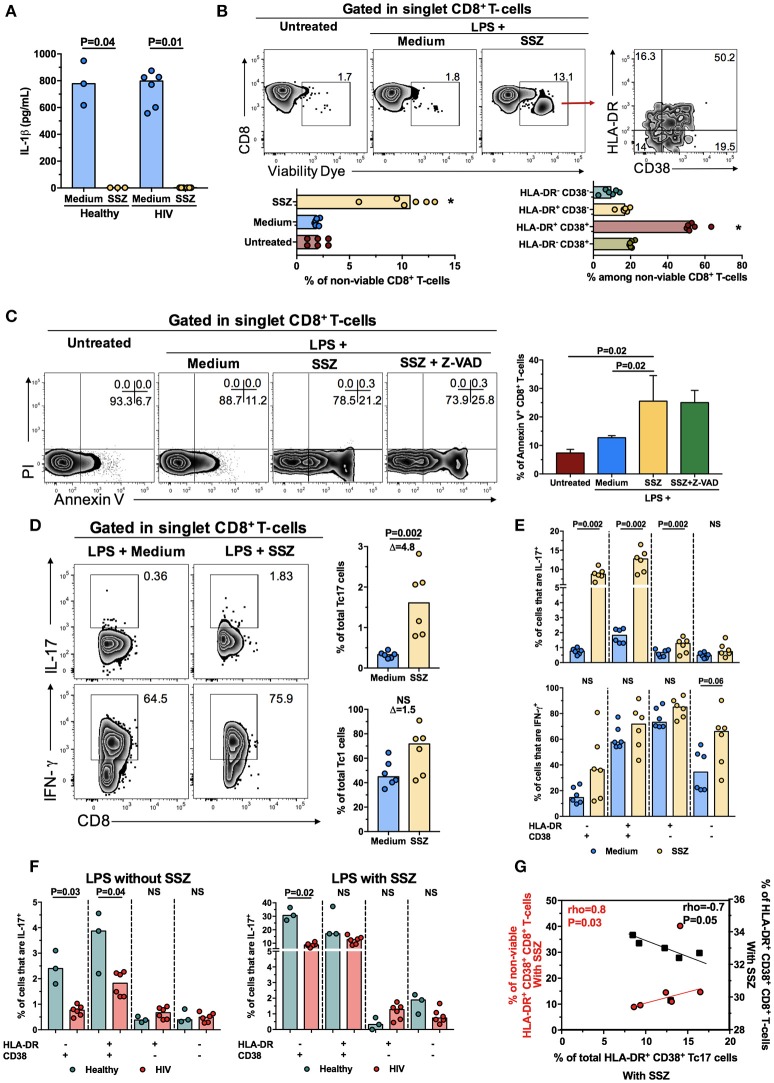
Sulfasalazine increases the frequency of total HLA-DR^+^ CD38^+^ Tc17 cells from PLWH. **(A)** Levels of IL-1β in culture supernatant from PBMC from healthy or PLWH treated with LPS in the presence of medium or sulfasalazine (SSZ). The *P*-value of the Wilcoxon test is shown. **(B)** Gating strategy for the detection of non-viable CD8^+^ T-cells in untreated and LPS-treated cells, the latter in the presence of medium or SSZ. The proportion of HLA-DR- and CD38-expressing non-viable CD8^+^ T-cells after SSZ treatment is shown in the right. Zebra plots from a representative subject living with HIV are shown. The summary of the frequency of total and HLA-DR- and CD38-expressing non-viable CD8^+^ T-cells in the respective conditions are shown. *n* = 3 healthy individuals and 6 PLWH. *P < 0.03 vs. all the other groups. **(C)** Gating strategy for the detection of Annexin V and/or Propidium Iodide (PI)-positive CD8^+^ T-cells in untreated and LPS-treated cells, the latter in the presence of medium, SSZ and SSZ plus Z-VAD. Zebra plots from a representative subject living with HIV are shown. The summary of the frequency of Annexin V^+^ CD8^+^ T-cells in the respective conditions is shown on the right. *n* = 4 PLWH. In **(B,C)** the *P*-value of the Kruskal-Wallis and Dunn's *post-hoc* test are shown. **(D)** Frequency of total Tc17 and Tc1 cells from a subject living with HIV after LPS treatment in the presence of medium or SSZ. The numbers in the zebra plots indicate the percentage of the populations. The summary of the results is shown at the right. The Δ of the SSZ/medium-treated cells frequency is shown at the top. **(E)** Frequency of IL-17^+^ (upper panel) and IFN-γ^+^ (lower panel) cells among HLA-DR- and/or CD38-expressing PMA-ionomycin-stimulated CD8^+^ T-cells from PLWH, after LPS treatment in the presence of medium or SSZ. In **(D,E)** the *P*-value of the Wilcoxon test is shown. **(F)** Frequency of IL-17^+^ cells among HLA-DR- and/or CD38-expressing PMA-ionomycin-stimulated CD8^+^ T-cells from healthy individuals and PLWH, after LPS treatment without (left panel) or with (right panel) SSZ. The *P*-value of the Mann-Whitney test is shown. **(G)** Correlation between the frequency of non-viable (red circles) and total (black squares) HLA-DR^+^ CD38^+^ CD8^+^ T-cells and that of IL-17-producing HLA-DR^+^ CD38^+^ CD8^+^ T-cells, all of them after LPS treatment in the presence of SSZ. The rho and *P*-value of the Spearman test are shown. NS, Not statistically significant.

The anti-inflammatory effect of SSZ was confirmed by the decrease of IL-1β levels in cell culture supernatant in comparison with the untreated condition (Figure 5A), as this agent blocks the activation of the nuclear factor (NF)-κB triggered by LPS-TLR4 interaction (30), on which IL-1β transcription is dependent (31). Next, we evaluated if SSZ induces the death of activated CD8^+^ T-cells. Although the mortality of total PBMC from PLWH after the SSZ treatment was <1%, when CD8^+^ T-cells were analyzed separately, they had a median (range) of mortality of 10.7% (9.5–12.3), which was significantly higher than in cells without SSZ treatment (Figure 5B). Indeed, among total non-viable PBMC after SSZ treatment, CD3^+^ CD8^+^ cells had the highest proportion [median [range] of 49.9% [36.8-61.8]], followed by CD3^+^ CD8^−^ [median [range] of 22.2% [18.5–28.3]] and CD3^−^ CD8^−^ cells [median [range] of 12.9% [6.2–27.2]]. Similar results were obtained in PBMC from healthy individuals (data not shown). Interestingly, among total non-viable CD8^+^ T-cells after SSZ treatment, HLA-DR^+^ CD38^+^ cells had the highest proportion, followed by HLA-DR^−^ CD38^+^ and HLA-DR^+^ CD38^−^ cells (Figure 5B). To explore the type of cell death induced by SSZ, PBMC from PLWH were cultured with LPS plus SSZ in the presence or absence of the pan-caspase inhibitor Z-VAD or the caspase-1 inhibitor Y-VAD, and the frequency of death CD8^+^ T-cells was evaluated by the binding of Annexin V and internalization of propidium iodide (PI), respectively. As shown in Figure 5C, SSZ particularly induced death of CD8^+^ T-cells, which was higher compared with cells without SSZ treatment. Interestingly, the CD8^+^ T-cells death was not prevented by Z-VAD or Y-VAD treatment (Figure 5C and data not shown). Again, HLA-DR^+^ CD38^+^ and HLA-DR^−^ CD38^+^ cells had the highest proportion among total Annexin V^+^ CD8^+^ T-cells, which have preserved membrane integrity and low non-specific binding of stain antibodies (*P* = 0.02, data not shown), confirming our results using the viability dye. Thus, SSZ particularly induced caspase-independent death of activated CD8^+^ T-cells.

Finally, we analyzed the effect of SSZ in the frequency of Tc17 cells. Interestingly, a significantly higher frequency of total Tc17 cells was found after SSZ treatment in comparison with untreated cells, both in PLWH and healthy individuals (Figure 5D, upper panels and data not shown). In addition, although SSZ also increased the frequency of total Tc1 cells, it was not statistically significant (Figure 5D, lower panels). Similarly, the frequency of total HLA-DR^−^ CD38^+^, HLA-DR^+^ CD38^+^, and HLA-DR^+^ CD38^−^ Tc17, but not Tc1 cells, increased after SSZ treatment, both in PLWH and healthy individuals (Figure 5E and data not shown). Remarkably, similar to our previous results (Figure 3E), the frequency of total HLA-DR^+^ CD38^+^ Tc17 cells from PLWH was lower than those from healthy individuals in the presence of LPS plus medium (Figure 5F, left panel), but it reached levels similar to those from healthy individuals in the presence of SSZ (Figure 5F, right panel). Of note, this effect was not evidenced in HLA-DR^−^ CD38^+^ Tc17 cells from PLWH, which despite their higher response after SSZ treatment (Figure 5E, upper panel), maintained lower frequencies in comparison with cells from healthy individuals (Figure 5F), confirming the negative effect of CD38 in the Tc17 cells response.

Ultimately, we hypothesized that the SSZ-induced increase of total HLA-DR^+^ CD38^+^ Tc17 cells in PLWH was associated with the preferential death of HLA-DR^+^ CD38^+^ CD8^+^ T-cells. Certainly, after SSZ treatment, the frequency of total HLA-DR^+^ CD38^+^ Tc17 cells was positively correlated with the percentage of non-viable HLA-DR^+^ CD38^+^ CD8^+^ T-cells, but negatively correlated with the frequency of HLA-DR^+^ CD38^+^ CD8^+^ T-cells (Figure 5G), consistent with an increase of Tc17 cells concomitant with the death and the decrease of activated CD8^+^ T-cells. Of note, the MeFI of HLA-DR or CD38 in HLA-DR^+^ CD38^+^ Tc17 cells were similar between SSZ-treated and untreated cells (P≥0.3, data not shown), indicating that the death of activated cells, but not the modulation of the expression of these activation markers is the mechanism responsible for the increase of the Tc17 cells frequency. In summary, through the decrease of the inflammatory environment and induction of death of activated –and possibly dysfunctional- CD8^+^ T-cells, SSZ increased the frequency of HLA-DR^+^ CD38^+^ Tc17 cells from PLWH.

## Discussion

Although their main localization is the gut and liver, circulating CD161-expressing and IL-17-producing T-cells have also been described (32, 33). Similar to a previous report (10), we found a decreased frequency of circulating CD161-expressing CD4^+^ and CD8^+^ T-cells, as well as a lower expression of CD161, in PLWH despite HAART-induced viral suppression. Although we did not evaluate, for instance, the Vα7.2-Jα33 T-cell receptor (TCR), which characterizes mucosal associated invariant T (MAIT) cells, the CD161-expressing CD8^+^ T-cells (and particularly CD161^hi^ cells) most likely constitute this population (34, 35), since MAIT cells are characterized by high CD161 expression (36), production of IL-17 (37) and are depleted in PLWH (38). In addition, this population is also susceptible to activation-induced cell death (38) and to CD161 downregulation (39), particularly induced by microbial products, which are often in circulation during HIV infection; this is consistent with the lower MeFI and frequency of CD161-expressing T-cells observed in our study. In addition, the increased microbial translocation could induce the traffic of CD161-expressing CD8^+^ T-cells from blood to gut mucosa (40). Certainly, the loss of CD161-expressing CD8^+^ T-cells is associated to HIV progression, evidenced by the preservation of their frequency in HIV elite controllers (41). Additionally, similar to total CD8^+^ T-cells, the expression of HLA-DR and CD38 is increased in CD161^+^/MAIT-cells from untreated PLWH (42). However, CD161-expresing CD8^+^ T-cells from our cohort of treated PLWH exhibited similar activation levels to those from healthy controls, indicating a HAART-induced normalization of these parameters.

Since CD161 has been used as a marker of IL-17-producing T-cells, we hypothesized that the frequency of total Tc17 cells was also decreased in PLWH. Nonetheless, the frequency of total Tc17 cells was comparable between PLWH and healthy controls, similar to that of Tc1 cells. This finding was associated to the downregulation of surface CD161 in CD8^+^ T-cells after polyclonal stimulation, as previously reported (39, 42), which hindered the previously established association between CD161 expression and IL-17 production in healthy donors (32). Of note, although in our experimental design, CD161 was dramatically down-regulated after polyclonal stimulation, CD161^+^ cells sorted from peripheral blood from healthy donors exhibit a high expression and secretion of IL-17, compared with CD161^−^ cells (9, 32). Indeed, the production of IL-17 is dominated by CD161^hi^ but not CD161^int^ CD8^+^ T-cells (32, 33), indicating that this subset is the predominant Tc17 population.

Interestingly, the production of IL-17 by total CD8^+^ T-cells was associated with the expression of HLA-DR and CD38, populations that had an increased frequency but lower functionality in PLWH. This finding is similar to a previous report, where dysfunctional CD8^+^ T-cells, particularly in their degranulation ability, have been observed in patients on HAART (43). Strikingly, HIV-specific Tc17 cells were preferentially HLA-DR^+^ CD38^+^ cells, suggesting that these cells could be also dysfunctional, as previously reported (3). In addition, a preferential production of IFN-γ over IL-17 by total HLA-DR^+^ CD38^+^ CD8^+^ T-cells was found in PLWH, in agreement with the depletion of circulating Tc17 but not Tc1 cells during chronic lentiviral infections (44). Nonetheless, the HIV-specific Tc17/Tc1 cells ratio was close to one in HLA-DR and/or CD38-expressing CD8^+^ T-cells, indicating that in HIV-specific cells is maintained a similar proportion between both profiles. It remains to be defined if this is similar for other antigen specificities. Moreover, the ACCENSE analysis showed a relationship between the expression of IL-17 and IFN-γ in CD8^+^ T-cells, particularly in PLWH, that was not evidenced by the detection of IL-17^+^ IFN-γ^+^ CD8^+^ T-cells by multiparametric flow cytometry. This is consistent with the ability of t-SNE to identify small cellular subsets and associations of markers at a single-cell resolution that might be missed with conventional cytometry gating (45). These IL-17^+^ IFN-γ^+^ CD8^+^ T-cells could reflect the shift from the Tc17 to the Tc1 profile in PLWH; in fact a similar shift has been previously reported to be induced by IL-12 in mice (46). Overall, the dysfunction of Tc17 cells could predispose PLWH to microbial co-infections. In fact, low plasma IL-17 levels were associated with mortality in PLWH (47).

Here we found that CD8^+^ T-cells from PLWH are prone to activation after *ex vivo* polyclonal stimulation. Whereas the co-expression of HLA-DR and CD38 has been associated with high activation and dysfunction of CD8^+^ T-cells, the single expression of each marker has a differential significance. Thus, HLA-DR^+^ CD38^−^ CD8^+^ T-cells have a better survival, higher polyfunctionality, cytotoxicity and proliferative ability than HLA-DR^+^ CD38^+^ CD8^+^ T-cells, and are preferentially seen in HIV controllers (8, 48, 49). On the other hand, CD38^+^ CD8^+^ T-cells are prone to apoptosis (3) and are associated with residual viral replication in patients receiving anti-retroviral therapy (50). In agreement with the dysfunction of CD38-expressing CD8^+^ T-cells, dysfunctional HLA-DR^+^ CD38^+^ Tc17 cells from PLWH had a higher expression of CD38, and the predominance of more functional HLA-DR^+^ CD38^−^ CD8^+^ T-cells was associated with a preserved IL-17 response. A major concern is that most of the HIV-specific CD8^+^ T-cells express CD38 (3, 51). Strikingly, the mechanism associated to CD38-induced dysfunction in CD8^+^ T-cells is still undefined. CD38 is an extracellular ADP-ribosyl cyclase which induces the conversion of nicotinamide adenine dinucleotide to cyclic ADP-ribose (cADPR), a molecule involved in the mobilization of calcium stores, critical for cell activation. However, CD38 is also a cADPR hydrolase, regulating its levels (52). Thus, it is possible that in the context of persistent immune activation, CD38 has a preferential cADPR hydrolase activity, impairing the functional ability of CD8^+^ T-cells. In addition to chronic activation, CD8^+^ T-cells from PLWH are also characterized by an exhaustion state, particularly evidenced by the expression of PD-1 (4), a receptor that blocks the TCR signaling (53). Interestingly, the CD38/PD-1 co-expression is observed in most of HIV-specific CD8^+^ T-cells, and has been positively correlated with viral load in untreated PLWH (54). Thus, the negative effect of CD38 and PD-1 could induce a greater dysfunction of CD8^+^ T-cells in PLWH, as we observed for total HLA-DR^+^ CD38^+^ Tc17 cells. Together, ours and previous reports indicate that CD38 and the concomitant expression of PD-1 are major contributors to the dysfunctionality observed in total and antigen-specific CD8^+^ T-cells in PLWH, underscoring the need for therapeutic strategies that target these molecules.

Despite the suppression of viral replication, HAART did not restore the frequencies of circulating CD161-expressing and HLA-DR^+^ CD38^+^ Tc17 cells, so that, in addition to viral replication, the persistent inflammatory environment driven by microbial translocation could be responsible for the alterations in these CD8^+^ T-cell subsets (55). A longer treatment could also be required for their normalization, as found for other immunological markers (56) and in our case for HLA-DR^+^ CD38^+^ Tc17 cells. In addition, the negative correlation between the percentage of HLA-DR^+^ CD38^+^ Tc17 cells, the HLA-DR^+^ CD38^+^ Tc17/ Tc1 cells ratio and plasma sCD14 levels in our cohort of PLWH suggest that this subset is affected by the persistent systemic activation; in addition, it might indicate that these alterations associated to low levels of Il-17 contribute to the systemic inflammation. Similar findings were obtained for Th17 cells in simian immunodeficiency virus (SIV)-infected macaques (57) and PLWH (58). Importantly, the frequency of HLA-DR^+^ CD38^+^ Tc17 cells could constitute a novel cellular marker of immune activation and/or reconstitution in patients receiving HAART. Of note, we did not evaluate other functional parameters in Tc17 cells, such as proliferation or secretion of other cytokines. In this regard, a previous study demonstrated a depletion of IL-17^+^ IFN-γ^+^ CD8^+^ T-cells in chronic SIV-infected macaques, suggesting a defect in this population during this lentiviral infection (44). However, to our knowledge, there are no reports that have evaluated the proliferative capacity of Tc17 cells in PLWH. Thus, it remains to be determined if other functional capacities are altered in Tc17 cells from PLWH.

Together, our results agree with an immune unbalance and CD8^+^ T cells dysfunction in PLWH despite suppressive HAART, which could contribute to morbidity or therapy failure in scenarios of T cell activation, such as co-infections and cardiovascular disease. Since systemic inflammation is an important cause of morbidity in these individuals (59), a major focus of research is the adjuvant administration of anti-inflammatory agents that reduce, among other parameters, the expression of HLA-DR and CD38 in CD8^+^ T-cells (17). In this sense, SSZ could be a useful immunomodulatory strategy, particularly in patients with inflammatory comorbidities. In addition to the decrease of inflammatory cytokines via inhibition of the NF-κB pathway, SSZ induces caspase-independent death of activated -and possibly dysfunctional- CD8^+^ T-cells, via the mitochondrio-nuclear translocation of the apoptosis-inducing factor (AIF), that induces DNA fragmentation (24, 60). These effects led to an increase of the frequency of total HLA-DR^+^ CD38^+^ Tc17 cells in PLWH to similar levels found in healthy controls. Interestingly, SSZ-induced cell death was preferentially observed in HLA-DR^+^ CD38^+^ and HLA-DR^−^ CD38^+^ CD8^+^ T-cells, in agreement with the lower expression of the anti-apoptotic protein Bcl-2 in CD38-expressing CD8^+^ T-cells (8, 61). Indeed, Bcl-2 prevents the SSZ-induced, AIF-mediated cell death (60). These mechanisms also explain the beneficial effects of SSZ in the treatment of chronic inflammatory disorders (21). Of note, it remains to be determined the if SSZ treatment, through the decrease of NF-κB-dependent cytokines such as IL-1β, IL-6, and IL-23, affects Th17 or Tc17 cells polarization, as these cytokines are important for their differentiation. Future studies that explore the mechanisms of SSZ in the restoration of particular CD8^+^ T-cell populations are needed. Moreover, clinical trials that evaluate HAART and SSZ co-treatment are required to establish the usefulness of this approach for decreasing the systemic immune activation state and the function of Tc17 cells in non-viremic PLWH.

## Author contributions

FP-C, MF, NT, and MR conceived the study. FP-C performed the experiments. MF optimized the conditions for SSZ *in vitro* treatment. FP-C, MF, NT, and MR analyzed the results and wrote the manuscript.

### Conflict of interest statement

The authors declare that the research was conducted in the absence of any commercial or financial relationships that could be construed as a potential conflict of interest.
